# Determinants of Work-Related Risks among Veterinary Clinical Students in South West Nigeria

**DOI:** 10.1155/2020/2780378

**Published:** 2020-08-01

**Authors:** Oluwawemimo O. Adebowale, Monsurat O. Afolabi, Hezekiah K. Adesokan, Olubunmi G. Fasanmi, Olanike K. Adeyemo, Olajoju J. Awoyomi, Folorunso O. Fasina

**Affiliations:** ^1^Department of Veterinary Public Health and Preventive Medicine, Federal University of Agriculture, Alabata, Abeokuta, Ogun State, Nigeria; ^2^Department of Veterinary Public Health and Preventive Medicine, University of Ibadan, Ibadan, Oyo State, Nigeria; ^3^Federal College of Animal Health and Production Technology, Ibadan, Oyo State, Nigeria; ^4^ECTAD, Food and Agriculture Organization of the United Nations (FAO), Dar es Salaam, Tanzania; ^5^Department of Veterinary Tropical Diseases, University of Pretoria, Pretoria, South Africa

## Abstract

Veterinary practices or activities expose professionals, including students, to hazards associated with animal contact. To describe workplace health and safety status and risk factors associated with hazards among veterinary clinical students in South West Nigeria, a cross-sectional survey was conducted using a semistructured questionnaire. Data on demographics, health and safety (HS) status, work-related hazards, healthcare facilities, and immunisation history were obtained. Of 167 students recruited, 100 (60.2%) were males, and >77.1% fell within the age group of 21–25 years. Many participants (77.0%) reported the lack of active HS committee. Exposures to various physical hazards (PHs) such as needlestick injuries (NSIs, 41.5%), animal scratches (42.0%), animal kicks (33.0%), falls/slips (25.0%), and, less frequently, animal bites (13.8%) were reported. Allergies (35.9%) and acute gastrointestinal infection (25.6%) mainly after contact with dogs presented with parvoviral enteritis were reported. For chemical hazards, 27.8% and 29.0% of participants indicated having had eye burn and choke on exposure to formalin. No adequate immunisation against either tetanus, rabies, or both was provided (<18%). An association between accommodation type and students' level of health and safety training was observed (OR = 0.46, 95% CI: 0.241–0.897, *p*=0.02), and frequencies of student contact with various animal types were strongly associated with exposures to different physical and biological risks (*p* < 0.05). This study revealed poor health and safety training, practices, and increased exposure of students to a wide range of hazards. Therefore, the development of mitigation programmes in veterinary schools becomes critical to safeguard students' wellbeing.

## 1. Introduction

Workplace health and safety focuses on primary protection of workers' health from hazards through preventive and proactive approaches including risk assessment, hazard identification, hazard mitigation, hazard elimination, and treatment of work-related injuries [1]. On the average, work-related accidents and illnesses kill more than four people every minute; and globally approximately two million workers die annually from occupation-related injuries and diseases [2, 3], with an estimated annual cost > $2.8 trillion [3]. Unfortunately, workplace health and safety remains neglected in many low-to-middle-income countries (LMICs), primarily due to the overwhelming social, economic, and political challenges, competing interest, and weak translational mechanisms for scientific evidence and facts into policies [4].

The veterinary field includes diverse professionals who interact with a variety of animals and work in the environment associated with occupational risks (physical, chemical, and biological hazards) [5, 6]. Injuries, trauma, and zoonoses remain the main occupational risk for veterinarians today, but new emerging risks, such as psychological risks, are becoming increasingly important for these workers [7]. Physical threats may arise from work equipment, motor accidents, and directly from patients (animals), usually in the forms of animal bites, scratches, kicking and fractures, crushing, bruising, and needlestick injuries [8]. Chemical/radiological hazards are primarily due to contact with disinfectants, anaesthetic gases, X-rays, pesticides, formaldehyde, antibiotics, and corrosive, oxidative, and chemotherapeutic agents [9]. These substances are implicated in cancer development, foetal losses, and/or congenital disabilities and deformities [10–12]. Biological hazards principally are exposure to zoonoses [13]; zoonoses account for ≥61% of all human contagions worldwide, including approximately 75% of emerging human diseases [14]. These diseases also cause serious health hazards among animal populations with attendant economic and public health consequences [15]. Disease impacts and costs can be highly variable depending on severity [16]. The first documented evidence of work-related zoonoses among veterinary students dated back to 1939, when infections caused by *Erysipelothrix* species were reported among students (*n* = 4) who got injured during horse dissection exercises [17]. A recent review had also described occupationally acquired diseases among veterinary students, including individual cases and outbreaks involving various zoonotic etiological agents [5].

In Nigeria, limited studies have described the various exposures and/or hazards or risks linked to veterinary practice, especially among veterinarians and veterinary students. The underreporting of hazards among these groups could be associated with inadequate surveillance and risk assessments at the workplace as well as a lack of comprehensive reporting systems. A previous survey on the hazards exposures of workers of animal-related occupations (including veterinarians) in Abeokuta, South West Nigeria, reported a prevalence of 69.6% occupational hazard exposures to a myriad of occupational specific and nonspecific hazards [18]. Some of the common hazards reported in the study included exposures to skin diseases, respiratory diseases and salmonellosis, animal/dog bites, animal kicks, and birds' pecking and scratching [18]. Also, brucellosis, which is an occupationally acquired zoonosis, has been reported majorly to infect veterinarians but not the students in Nigeria [19].

The current occupational health and safety among veterinarians and veterinary students in Nigeria are unknown. There is also paucity in previously documented injuries, exposures, and/or hazards or risks among veterinary students in Nigeria. To the best of our knowledge, this study is the first work to provide data on the workplace health and safety status in the two veterinary schools in South West Nigeria (SWN) and the associated risks among veterinary clinical students.

## 2. Methods

### 2.1. Study Location

**Figure 1:**
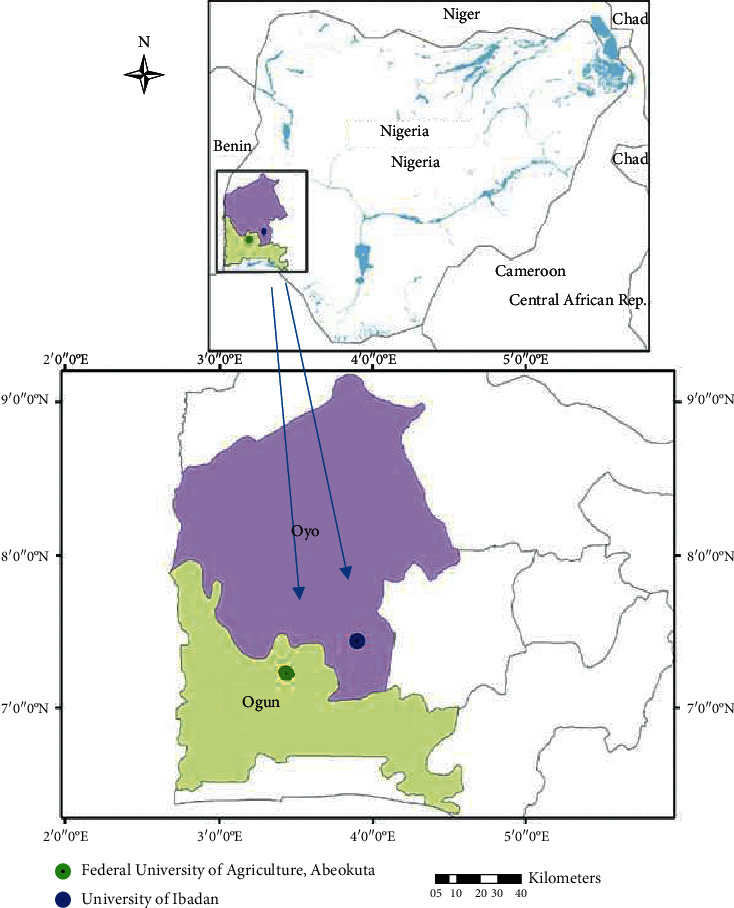
A spatial representation of the university locations of the students who participated in this study.

The study was conducted among the two veterinary schools at the Federal University of Agriculture, Abeokuta, Ogun State, Nigeria (FUNAAB, Vet School 1) and the University of Ibadan, Ibadan, Oyo state (UI, Vet School 2).  Figure 1 shows a spatial representation of university locations of participating students. The Federal University of Agriculture, Abeokuta and the University of Ibadan have estimated populations of 19,000 and 47,100, respectively (comprising students and members of the academic and administrative staff). The veterinary schools within the respective institutions have approximately total student populations of 216 and 527. Ogun and Oyo States have projected populations estimated at 5,217,716 and 5,580,894 residents, respectively, according to the National Population Commission (NPC) and National Bureau of Statistics [20]. Ogun is bordered in the south by Lagos State, in the north by Oyo and Osun States, in the east by Ondo, and in the west by the Republic of Benin, while Oyo state is bounded north by Kwara State, in the east by Osun State, in the south by Ogun State, and in the west partly by Ogun State and partly by the Republic of Benin. The veterinary schools within the studied institutions provide various clinical and diagnostic services to a wide range of animals, which include food animals (cattle, sheep, goat, pigs, and poultry), small animals (dogs and cats), wildlife, horses, and donkeys.

### 2.2. Study Design and Questionnaire Administration

A cross-sectional study was conducted among veterinary clinical students (5th and 6th years) in the two veterinary schools in South West Nigeria from May to December 2017 as the students at these levels have similar exposures at training. Students in the preclinical and paraclinical years (2nd to 4th years) were excluded as these latter group of students do not have intense contact with animals yet and the appreciation of health and biosafety is expected to be little. To ensure internal consistency (reliability), avoid ambiguity, identify response options, and estimate likely duration of each interview using the measuring instrument, the questionnaire was pretested among thirty-five undergraduate DVM III (400 level) students from FUNAAB [21] who were not included in the study. The questionnaire was thereafter modified based on the feedback received from the pretest. The survey tool was designed to include both open- and closed-ended questions drawn from the reviews of previous papers relating to the subject.

The questionnaire was subdivided into four sections, viz, (1) demographic data of participating students (7 items): *age, gender, religion, marital status, year of entry into veterinary schools, type of accommodation provided, and reasons or motivation for studying veterinary medicine*; (2) health and safety training received or being received during veterinary degree programme (12 items); (3) information on student safety practices and injuries or illnesses exposed to during formal veterinary training. To determine the hazards veterinary clinical students were frequently exposed to, multispecies animal-level data were gathered including the frequency of contact, exposure to physical, biological and chemical hazards, and if such were reported to appropriate supervisors or health care centres (25 items); and (4) specific questions on the provision of health care facilities, pre- or postexposure immunisation status, especially against tetanus and rabies (6 items).

The authorities of the two schools of veterinary medicine investigated in this study were informed about the survey, and letters with signed consent and authorisation to carry out the research were provided. The inclusion criteria for the participation of the students were that students must be fully registered in the veterinary schools of study and clinical years designated as DVM IV (5th year) and DVM V (6th year). Meetings were held with eligible students (213) and informed on the purpose of the study, the content of the questionnaire, and that participation was entirely voluntary, and that no monetary incentives were involved. Students were asked if they understood the purpose of research and involvement before they were requested to give their informed consent and fill the questionnaire. Participants were also aware of their right not to fill in the questionnaire or some questions within the questionnaire or disengage from the study at any period during the process without providing any reason without any attached penalty. No parents' consent was required, as all students were above the legal age. Selection of the participants was based on willingness to participate in the study. All procedures implemented during the data collection enabled confidentiality and anonymity with no uniquely identifiable data collected. All consenting participants were included in the study and the questionnaire was self-administered. Questionnaire administration took between 20 and 25 minutes per participant.

### 2.3. Data Analysis

Data generated were captured and filtered in Microsoft Excel^®^, 2013 (Microsoft Corporation, Redmond, WA). Data analyses were conducted by Graph Pad Prism 8.0.0 (descriptive statistics and figure presentations) and Stata 12.0 (inferential statistics). Anonymity and data's confidentiality were guaranteed throughout the study. Binary responses were captured as follows: “*Yes*” and correct answers to questions on health and safety training, exposure and biosafety practices as well as health care were scored “1” and “*No*” and incorrect responses were scored as “0.” Categorical data were summarised as frequencies and percentages. A cut-off score of ≥70% was regarded as high health and safety training, exposure and biosafety practices as well as health care levels. Using the two-by-two table, the bivariate analysis was carried out with the threshold cut-off value set at *p* ≤ 0.05 for Pearson's Chi-square (*χ*^2^) in Fisher's exact test. All variables significant at the 25% level with the main outcome measures in the bivariate analysis were included in the logistic regression model. The decision for liberal *p* value at this step was to ensure that crucial potential predictor/risk variables were included in the model [17]. All retained variables at the univariable analysis were tested in logistic regression and considered significant at a probability cut-off of 0.05. Odds ratios (OR) were computed to determine the presence and strength of the associations between variables, and 95% Confidence Intervals (CIs) were calculated to investigate the statistical significance for each predictor variable.

## 3. Results and Discussion

### 3.1. Participants' Characteristics

**Figure 2:**
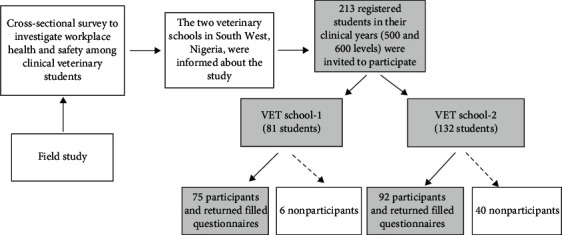
Flowchart process for recruitment of respondents from the two veterinary schools in South West Nigeria between May and December 2017. In Nigeria, clinical students (500 and 600 levels) are designated as DVM IV and DVM V, respectively.

This study has provided fundamental information on occupational health and safety status as well as risk factors associated with poor compliance with safety standards among veterinary students during their formal training programme in the two veterinary schools in South West Nigeria. The recruitment flow diagram is presented in Figure 2. A total of two hundred and thirteen questionnaires were administered, out of which 167 were filled and returned, giving a response rate of 78.4%. Approximately 47.3% (79/167) of participants reported residing on campus, while 52.7% (88/167) lived outside the school premises. Only 33.5% (56/167) of the participants were motivated to study veterinary medicine because of their passion for animals. The majority of the participants were males (60.2%) and the dominant age group was 21–25 years (77.1%). More male participants than female were involved in the study, a reflection of the male domination in the veterinary profession in low-middle-income countries (LMICs), particularly in Africa. However, this trend is changing globally, especially in the Western world [22–25] and in South Africa [26].

### 3.2. Health and Safety Training, Biosafety Practices, and Hazard Exposure

Concerning information relating to health and safety training, 80.8% of participants were not aware of the presence of a committee in the school (135/167). More than 65% of the participants confirmed the fact that they did not receive health and safety training, with less than 40% competence to work safely. The participants (51.1%) likewise indicated that work environments were unsafe for them, and prompt responses of school management to investigate any identified hazards and formal system for injury reporting and documentation were adjudged inadequate. The observed inadequacy in health and safety (HS) and work conditions is not surprising, as issues relating to this are becoming a concern in Nigeria, with many institutions lacking adequate operational health and safety systems and training, risk assessments, and hazard reporting protocol [27]. The Labour, Safety, Health and Welfare Bill of Nigeria (2012) empowers the National Council for Occupational Safety and Health in Nigeria to enforce and implement well-maintained environment to ensure the psychological, physical, and social wellbeing of workers [28]. Contrastingly, the occupational health and safety regulations, though well documented, are weakly implemented and enforced to date [28]. Intensified training and awareness of HS at workplaces should be strengthened for students' internalisation/acculturation and adoption.

The format for health and safety training was observed to be mainly by didactic theoretical teachings. The various communication resources, i.e., didactic lessons, practical/clinical rotations, and online or manuals, were suitable for health and safety training or awareness for the students, without any significant difference (*p* > 0.05). Although online training on HS will be apt and cheaper to deliver student training, countries like Nigeria is yet to achieve the level of the Internet efficiency and power supply infrastructure to support the same; it behoves the authority to invest in these critical areas to intensify HS training.

Furthermore, no fire hazard training or drills have ever been provided, and less than half (<34%) knew the specific steps to take in an outbreak situation. Material Safety Data Sheet (MSDS) was also hardly used (39.5%; 66/167). Based on participants' perception, training on the use of personal protective equipment (PPE), potential hazards/risks related with the profession, safe handling of animals including large and small animals, handling and disposal of sharp objects (such as needles), and biological and chemical substances was adequate (>90%). The univariable analysis showed an association between the sex of participants and the level of exposure to hazards (*p*=0.14) at a 25% level of significance (Table 1). However, logistic regression further indicated that male participants were about 4.76 times more likely to have a high risk of hazard exposure (OR = 4.76, 95% CI: 0.485–46.79; *p*=0.18) than females although not significant at 5% significant level (Table 2). The veterinary profession is one of the common male-dominated occupations in Nigeria which has higher numbers of male professionals and students. More so, males tend to dare or take risks more than females. These may explain the reason for being at higher risks of hazard exposure than female counterparts. Meanwhile, a significant association existed between students' types of accommodation and level of health and safety training based on the logistic regression. Specifically, residence on campus lowers the odds of health and safety training (OR = 0.46, 95% CI: 0.241–0.897, *p*=0.02; Table 2). Practically, students often get engaged in on-the-training job by understudying senior veterinary professionals on the field. As such, those residents outside the campus were more likely to get connected to these professionals considering possible nearness of residence, enabling accessibility and more contact time. This might be responsible for the increased odds of health and safety training exposure among those respondents living off campus.

### 3.3. Various Hazards Experienced among Students during Contact with Animals

**Figure 3:**
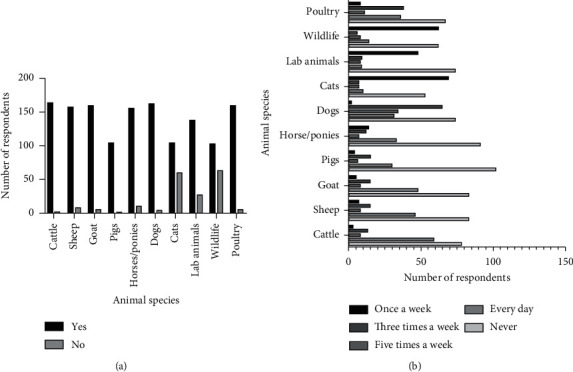
Various animal species and the frequency of contact by veterinary clinical students. (a) Animal types that veterinary students have been in contact with during their DVM programme. (b) Frequency of contact with the various animals once a week, three times a week, five times a week, every day, and no contact.

Participants reported having had interactions with at least one variety of animals or the other (Figure 3(a)).  Figure 3(b) shows the frequency of contact with the various animal species during clinical rotations. In this study, unsurprisingly, students have less contact with cats and wildlife than other animal species. Cats are rarely managed as pets in Nigerian homes because of cultural and traditional myths that associate “terrible luck” with cats. Also, the country currently has limited professional expertise in the wildlife field, inadequate facilities for the safe handling and restraints, and restricted diversity of wildlife species for student training.

**Figure 4:**
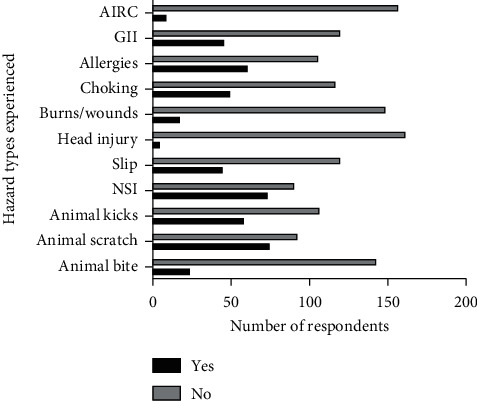
The different types of hazards experienced by veterinary clinical students during their professional training. AIRC = accidental ingestion of ruminal content; GII = gastrointestinal infection; and NSI = needlestick injury.

The physical forms of risks of injuries experienced by students during their various contact with animals in increasing order included falls/slips, animal kicks, needlestick injuries, and animal scratches. Meanwhile, eye burns and choking were the most common chemical risks suffered as a result of contact with formalin at anatomy practicals (Figure 4). The self-reported level of chemical exposures to formalin may be linked with a dearth of information or undervalued use of MSDS. The majority of participants confirmed they had no idea of the importance of MSDS. The MSDS contains the necessary information on chemical products and all hazardous materials and adequately describes the properties and potential hazards of the chemicals, as well as appropriate safety measures in terms of what PPE to use and steps to take in an emergency exposure.

For biological vulnerabilities, 27% (45/167) of the students supposedly reported suffering from gastrointestinal infection (GII) due to contact with animals and accidental ingestion of ruminal content (Figure 4). Twenty (20/162; 12.3%) diarrheal conditions associated with a dog contact were reported to be linked with confirmed parvoviral cases (11/20, 55%). Six participants linked GII to posthandling of bacterial cultures during microbiological procedures (30%). Students assessed indicated the handling of confirmed cases of canine parvoviral (CPV) and bacterial culture during microbiology rotations in their exposure to GII. Canine parvovirus is a highly contagious infection that spreads among dogs through the contaminated faeces. CPV is not categorised as affecting humans, and reports of acute gastroenteritis by the students after handling such cases are surprising. The claim by students could have been associated with the presence of cobacterial pathogens common with CPV which infect humans. For instance, several past studies have recorded multiple secondary bacterial enteropathogens shedding in dogs with CPV. Some of these pathogens include *Salmonella* spp., *Campylobacter* spp., *Clostridium* spp., and *Escherichia coli* [29, 30]. Furthermore, whether the students have had other underlying conditions that aggravated the risks were not explored in this study. Although students are provided with information on excellent laboratory and clinical practices (GLCP), noncompliance to these GLCP guidelines, poor hand washing practices, and inadequate provision of hygiene facilities may have contributed to GII experienced among the students.

Participants were worried about contracting zoonoses mostly rabies (67.9%, 112/165), bovine tuberculosis (62.3%, 104/167), and *Escherichia coli* O157 infections (60.4%, 99/164). Rabies was primarily a concern to students due to its attendant high fatality and transmission sources and inadequate immunisation against the infection. We observed that low numbers of students in year 6 (19% and 1%) indicated they received immunisation against tetanus and rabies, respectively, which was self-provided. There was no history of immunisations against tetanus or rabies according to the year 5 clinical students. Rabies is a known tropical neglected, but preventable disease both in humans and dogs [31]. An estimated 21,476 human deaths occur each year in Africa due to dog-mediated rabies [32]. A previous limited-scale assessment of veterinary medical students on clinic had established that only 3.3% (1/30) of the students have protective antibodies [33]. Whether the antibodies are in response to vaccination or previous history of dog bites was not established through DIVA strategy as none exist for rabies. Students may not have received immunisations against this disease for various reasons including lack of adequate provisions by institutions, the high cost of vaccines, and low perception of the public health risks.

### 3.4. Frequency of Animal Contact and Associated Risk Pattern

Different patterns of risks were observed: more animal contact increased the likelihood of exposure to hazards. For instance, participants in contact with wildlife five times a week have 26 times the odds of exposure to head injuries (95% CI: 2.05–329.68; *p*=0.01) compared with nonexposure. Contact with laboratory animals three times a week increased the odds of exposure to bites from laboratory animals (OR = 7.1; 95% CI: 1.54–32.71; *p*=0.01; Table 3) more than students who were not exposed. Furthermore, contact with horses five times a week and more increased the odds of exposure to slip or fall and gastrointestinal infections by 12.5 (95% CI: 1.76–88.74; *p*=0.01) and 5 times (95% CI: 1.05–23.79; *p*=0.04), respectively, compared to nonexposure. Similarly, contact with pigs three times a week or more increases the odds of student exposure to slip or fall (OR = 9.94; 95% CI: 1.15–86.06; *p*=0.04; Table 3) and needle prick injuries (OR = 9.00, 95% CI: 1.46–55.48; *p*=0.02) than noncontact. The frequent contact with sheep five times in a week increases student exposure to needle prick injuries as well (OR = 6.00, 95% CI: 1.17–30.72; *p*=0.03) and gastrointestinal infections (OR = 10.83; 95% CI: 1.37–85.44; *p*=0.02; Table 3) more than student without contact. Furthermore, odds of needlestick injuries increased as the frequency of contact with goats increased from three times (OR = 12.00; 95% CI: 1.44–99.67; *p*=0.02) to five times a week (OR = 36.00, 95% CI: 2.69–481.21; *p*=0.01).

While we reported here that the frequency of contact with various animal species increased the likelihood of veterinary students' exposure to hazards—mostly, physical animal-related injuries such as bites, scratches/bruises, and kicks—similar findings have been found elsewhere [9, 34]. Bites and scratches have been reported as regular injuries that contributed to the loss of workdays by veterinarians working with small animals (dogs and cats). Interestingly, this study did not associate any significant bite injuries with dog and cat contact, but with laboratory animals. It is noted that dog muzzle is often regularly sighted as restraint facility in the teaching clinics, and its regular use may be responsible for this observation.

Bites from laboratory animals were reported in a national survey conducted in the United States, in which, 15 out of 198 organisations surveyed indicated workers experienced animal bites [35, 36]. Nevertheless, little or no documentation exists on the risk arising from laboratory animals to veterinary professionals in Nigeria. Laboratory animals are relatively small; they need distinctive restraint facilities. Bites experienced by students may be influenced by individual perceptions and attitudes that increase noncompliance to standard laboratory animal handling protocols, complacency or negligence, and hence, the underestimation of risk. The magnitude and regularity of laboratory animal bite exposures among veterinary professionals in the country need further investigation.

Animal kicks and related injuries are familiar occurrences within the profession, especially among large animal workers, and the severity may range from minor to significantly life-threatening [9, 11, 25]. A study conducted in a city in Southwest Nigeria reported an overall 15% prevalence of animal kicks among animal-related occupations [18]. This prevalence was, however, not disaggregated by profession, and looking at the geographical scope of the study, the prevalence may be higher in certain professional categories than others. Considering the physical hazards like head injuries, slips/falls, students must take insurance against associated training risks, increase caution in animal handling and for authorities to approve facility designs that mitigate risks and avoid very narrow passageways that predispose staff and students to avoidable hazards.

Furthermore, data on the incidence of NSIs appeared high at 43.7% among the students in this study. This is lower than reports from previous studies conducted among veterinary professionals in Nigeria and other countries [37, 38]. The high incidence of NSI raises concerns because of the potential of adverse effects and transmission of diseases (iatrogenic and nosocomial) from an inadvertent injection of contents [36]. NSIs could arise from the lack of in-depth understanding of animal behaviours (ethology), improper handling, and poor restraint techniques of large animals. Pigs and goats tend to have the natural urge to attempt to escape, given the slightest space with a resultant increased risk of injuries to handlers.

## 4. Conclusions

Currently, there is a lack of formal health and safety committee in veterinary schools in South West Nigeria, which affects training-related practices and exposes students to risks. Previous studies showed a lack of attention to exposures to hazards among veterinary professionals in the veterinary workplace. Empirical evaluation of compliance with health and safety standards and risk factors are scanty in Nigeria. Hence, considering growing issues and the need for a strong policy in safety and health among veterinary students and professionals, the study becomes important to fill this gap. The study provided an empirical evaluation of students' level of health risks in the veterinary training facilities or workplace and observed that the risks of student exposure to hazards varied based on animal species worked with. We, therefore, recommend that training institutions must review the protocol of delivering clinical training in veterinary institutions and make compulsory safety standards like immunisations, mandatory training in health and safety, and hazard perceptions tutoring part of the programme in the veterinary curriculum. Development of risk assessment plans, health and safety guidelines, good practices, and mitigation systems to reduce workplace-related hazards must be carried out from training institutions to workplace postgraduation.

Certain limitations of the study are acknowledged. All the information provided by this study were voluntary and self-reported by student participants. Therefore, we cannot rule out any systematic biases inherent in the data. Also, this study was location bound; hence, external generalizability of our findings of the risks and exposures to other various veterinary schools across Nigeria may be limited.

## Figures and Tables

**Table 1:** Distribution of participants' demographics with the levels of health and safety training, exposure to hazards and biosafety practices, and access to health care facilities.

Variable	Category	Level of health and safety training received			Level of exposure to hazards and biosafety practices			Access to health care facilities			Total participants
		Low	High	*χ* ^2^; *p* value	Low	High	*χ* ^2^; *p* value	Low	High	*χ* ^2^; *p* value	
Sex (*n* = 167)	Female	21	45	1.00	4	62	1.00	23	43		66
	Male	34	67	0.06; 0.80	1	100	2.16; 0.14^*∗*^	34	67	0.03; 0.88	101
Age in years (*n* = 167)	≥25	14	22	1.00	1	35	1.00	9	27		36
	<25	41	90	0.74; 0.39	4	127	0.01; 0.93	48	83	1.70; 0.19^*∗*^	131
Accommodation (*n* = 167)	Off-campus	22	66	1.00	3	85	1.00	31	57		88
	On-campus	33	46	5.3; 0.02^*∗*^	1	78	0.82; 0.37	26	53	0.1; 0.75	79
Motivation (*n* = 167)	Not motivated	18	41	1.00	1	58	1.00	20	39		59
	Motivated	37	71	0.24; 0.62	3	105	0.19; 0.66	37	71	0.002; 0.96	108
Methods of training (*n* = 163)	No contact with trainer				1	47	1.00				48
	Contact with trainer	NA	NA	NA	1	114	0.41; 0.52	NA	NA	NA	115

#NA: not applicable. ^*∗*^Variables significant at *p* ≤ 0.25. High and low scores were set at ≥75% and ≤74.9%, respectively. Reference = 1.00.

**Table 2:** Logistic regression analysis of participants' demographics with the levels of health and safety training, exposure to hazards and biosafety practices, and access to health care facilities.

Variable	Category	Level of health and safety training received			Level of exposure to hazards and biosafety practices			Access to health care facilities		
		OR	95% CI	*p* value	OR	95% CI	*p* value	OR	95% CI	*p* value
Sex	Female				1					
	Male	—	—	—	4.76	0.49–46.79	0.18	—	—	—
Age in years	<25							1		
	≥25	—	—	—	—	—	—	0.58	0.25–1.33	0.20
Accommodation	Off campus	1								
	On campus	0.46	0.24–0.90	0.02^*∗*^	—	—	—	—	—	—

OR: odds ratio; CI: confidence interval. ^*∗*^Variables significant at *p* ≤ 0.05; reference = 1.00.

**Table 3:** Logistic regression analysis of participants' exposure to hazards with the frequency of contact with animals.

Animal	Hazard	Once a week			Three times			Five times		
		OR	95% CI	*p* value	OR	95% CI	*p* value	OR	95% CI	*p* value
Poultry	Needle prick	1.80	0.51–6.31	0.36	1.25	0.32–4.83	0.75	4.0	0.80–20.02	0.09
Wildlife	Head injuries	2.69	0.24–30.38	0.42	6.00	0.35–102.01	0.22	26.00	2.05–329.68	0.01^*∗*^
Laboratory animals	Bites	1.43	0.51–3.99	0.50	7.09	1.54–32.71	0.01^*∗*^	2.95	0.50–17.52	0.23
	Slip	3.55	1.51–8.32	0.004^*∗*^	1.90	0.34–10.64	0.46	4.00	0.81–19.69	0.09
Cats	Slip	2.38	1.07–5.25	0.03^*∗*^	3.17	0.80–12.53	0.10	11.88	2.11–66.73	0.01^*∗*^
Dogs	Scratch	0.12	0.02–0.82	0.03^*∗*^	0.79	0.13–5.01	0.80	0.40	0.06–2.45	0.32
Horse	Slip	1.5	0.46–4.88	0.50	2.50	0.69–9.12	0.17	12.50	1.76–88.74	0.01^*∗*^
	Gastrointestinal infections	2.11	0.66–6.76	0.21	1.11	0.28–4.47	0.88	3.75	0.59–23.66	0.16
Pigs	Needle prick	4.37	0.93–20.56	0.06	9.00	1.70–47.60	0.01^*∗*^	3.00	0.31–28.84	0.34
	Slip	4.22	0.53–33.90	0.18	9.94	1.15–86.06	0.04^*∗*^	13.00	0.98–172.95	0.05^&^
Sheep	Needle prick	2.39	0.62–9.12	0.20	5.20	1.29–20.94	0.02^*∗*^	6.67	0.99–45.04	0.05^&^
	Gastrointestinal infections	2.20	0.46–10.57	0.32	2.30	0.45–11.68	0.32	10.83	1.37–85.44	0.02^*∗*^
Goats	Needle prick	7.92	0.98–63.83	0.05^&^	12.00	1.44–99.67	0.02^*∗*^	36.00	2.69–481.21	0.01^*∗*^

OR: odds ratio; CI: Confidence Interval. ^&^Variables marginally significant; ^*∗*^variables significant at ^*∗*^*p* ≤ 0.05.

## Data Availability

The datasets used to support the findings of this study are available from the corresponding author upon request.
